# Bacterial adhesion and microleakage of restorative materials for sealing accessory canals in implant prosthesis

**DOI:** 10.6026/973206300200678

**Published:** 2024-06-30

**Authors:** Vishal Rathod, Satish Gujjarlapudi, Sridevi Kaul, Sneha Saraf, Deepak Sharma, Neetu Kharat

**Affiliations:** 1 Department of Prosthodontics Crown and Bridge, Faculty of Dental Science, Dharmsinh Desai University, Nadiad, Gujarat, India; 2 Familia dental, 804 S Green River road, Evansville, IN 47715; 3 Sterling Smiles, Nashua, NH, USA; 4 Department of Prosthodontics, Pacific Dental College and Research Center, Udaipur; 5 Military Dental Centre, Fatehgarh , Fatehgarh Military Station, Uttar Pradesh, India; 6 Department of Conservative Dentistry and Endodontics, RKDF College and Research Centre, Bhopal, India

**Keywords:** Accessory canals, screw retained implant supported prosthesis, microleakage, adhesion, restorative materials

## Abstract

The level of bacterial adhesion and bacterial microleakage in four different materials utilised to seal the access passage of screw retained implant supported prosthesis (SRIP) is of interest to dentists. Four distinct categories were created from the samples on the basis of restorative materials used for sealing access passage in SRIP. Guttapercha and light cured acrylic resin were found to have comparatively low bacterial adhesion and bacterial microleakage in sealing accessory canals in screw retained implant supported prosthesis.

## Background:

The primary benefit of screw-retained implant based prostheses (SRIP) over cement-retained ones is that the restorations can be removed as necessary [1, 2-3]. An additional advantage is lowering the chance of developing inflammation around implants owing to absence of cement requirement in (SRIP) [4, 5, 6- 7]. However, for SRIP to be stable and successful over the long term, an appropriate closure of the access channel is advised [5-8]. Malodor and the development of inflammation around dental implants have been related to pathological as well as non-pathological bacterial microleakage including bacterial proliferation of the internal implant structure[7-9]. These factors may ultimately result in implant failure due to bone loss. The efficacy of various materials in sealing the access passages of SRIP has been the subject of numerous investigations [10, 11- 12]. According to studies, the usage of wax, cavit, vinyl polysiloxane, gutta-percha, polytetrafluoroethylene (PTFE) tape, and other spacer materials was preferable to cotton pellets and endo-frost cotton pellets that have been shown to exhibit bacterial as well as fungal adhesion [13, 14, 15, 16-17]. The literature currently available suggests the superiority of using PTFE tape for securing the screw channel, primarily because it carries a lower load of microbiological density and volume, which has a significant impact on the implant system's long-term durability [18, 19, 20- 21]. Additional research revealed that PTFE tape was simpler to work with, sterilize, and retrieve when necessary [12-15]. Numerous studies have examined how well various materials cover the access passage of SRIP. Instead of focusing on the restorative materials that can be applied to mask the coronal portion of the screw-access opening, the majority of these investigations examined the efficacy of the inner filling materials [11-16]. A study measuring the microbial load that happened when spacer materials were used to seal the entire screw-access passage revealed that using gutta-percha or PTFE tape in conjunction with resin composite resulted in the lowest numbers of species of microbial organisms [13- 17]. The greatest percentage of microbial colonies, however, was linked to the combination of light-cured provisional composite with cotton pellet [16-19]. These days, composite resin constitutes materials most frequently utilized in dental restorative procedures. It has also been employed as the standard material for covering the coronal portion of the SRIP access passages [4- 8]. The main reasons composite resin products are employed are their excellent mechanical and aesthetic qualities [9-12].The main disadvantage, however, is the shrinking of the polymerization and the ensuing internal tensions, which may cause the creation of microcracks and bacterial microleakage [8-14]. When creating a temporary fixed prosthesis, one of the most often utilized materials in prosthodontics is polymethyl methacrylate acrylic (PMMA) [2- 6]. PMMA is an affordable material with strong marginal adaptability and polishing capabilities. However, wear vulnerability and elevated polymerization shrinkage are the primary drawbacks [7- 13]. Bisphenol Bis-GMA, or glycidyl methacrylate, was subsequently introduced for comparable uses [10-17]. According to the results of a comparison study, Bis-GMA material has better qualities than PMMA because it has greater marginal adaption, lower polymerization shrinkage, and lower bacterial microleakage [12-16]. The abrasion resistance of Bis-GMA is increased by the inorganic filler content. There is paucity of published data on the various restorative materials used to prevent enhanced bacterial penetration and proliferation by sealing the coronal portion of the access passage of SRIP [15-18]. Therefore, it is of interest to evaluate and compare bacterial adhesion and microleakage of four different materials (composite resin, light cured acrylic resin, Bis acryl and Gutta percha) utilised to seal the access passage of SRIP, while using PTFE tape as a spacer material.

## Methods and Materials:

The specimens investigated included 48 straight titanium abutments having a 4.5 mm diameter and a 3 mm cuff height, as well as 48 implant analogs that imitated dental implants. Four distinct categories were created from the samples on the basis of restorative materials used for sealing access passage in SRIP. Twelve samples were kept in each category. Applying a screwdriver, the titanium abutments were manually torqued to the analogs in an aseptic environment. After that, molds for auto polymerizing acrylic resin contained a portion of each analog. By applying a sterilized plugger, a 45-mm PTEE tape was bent and then packed. The restorative material from each group was placed into a 3-mm coronal height, assessed using a sterile UNC 15 periodontal probe.

Category P: Condensable composite resin used for sealing of access canal

Category Q: Light-cured acrylic resin (PMMA) used for sealing of access canal

Category R: Bis-acryl (Protemp^TM^ 4) used for sealing of access canal

Category T: Guttapercha used for sealing of access canal

## Thermocycling test:

To imitate temperature variations in the oral environment, the samples were submerged in a thermocycling device that alternated between both cold and hot bath tanks. A typical operating procedure was followed, with the machine configured for cold water baths at 5°C along with hot water baths at 55°C [3-9]. In each bath tank, a cycle lasted thirty seconds. For two weeks, the samples underwent 2000 cycles, or nearly 2.5 months intraoral [4-12]. Following the thermocycling test, each sample from every group had its restorative material condition assessed.

## Microbiology:

Two characters were used to code each sample: a number plus a letter (P,Q,R or T) that corresponded to the sample group in question. To ensure that every piece of restorative material was completely submerged in the contaminant solution containing Escherichia coli inside Luria-Bertani broth, the specimens were submerged individually in containers. For every sample at the 0.5 McFarland level, the DensiChek Plus device was used to standardize the baseline optical density. In order to guarantee that *E. coli* would proliferate through solution turbidity for the duration of the experiment, a positive control specimen was made under similar conditions. To validate the results, a negative control comparable specimen was utilized, and its transparency was verified under identical incubation conditions. After that, the specimens were incubated at 37°C for 7 days. After seven days, optical density was confirmed in every sample. The samples were double washed with phosphate-buffered saline to get rid of the not adhering bacterial cells before assessing the bacterial surface adhesion. Each sample's surface was sampled, and the inoculation was applied to an agar plate for an overnight growth period at 37°C. The specimen number, the corresponding group letter, and the symbol (S), which stands for surface swabs, were coded on each plate in following manner

Category P: SP1-SP12

Category Q: SQ1-SQ12

Category R: ST1-ST12

Category T: ST1-ST12.

Following the incubation phase, the covering restorations were taken away from the analogs while the PTFE tape remained intact. This was accomplished with a round carbide bur with a high-speed handpiece. After that, samples were taken with a micropipette from within the screw-access channel, and they were cultivated for 24 hours on nutritional agar and MacConkey mediums. Lastly, each specimen's total colony-forming units per milliliter (CFU/ml) were determined.

## Statistical analysis:

An IBM product from Chicago (USA), SPSS-20.0, was used to evaluate statistical data. The mean, standard deviation, median, and interquartile range were among the descriptive statistics used to present the numerical data of the bacterial counts. The two primary factors-average numbers of bacterial colonies and the proliferation of bacteria among the four categories (P, Q, R and T) were compared using the Kruskal-Wallis test. Later, this numerical variable was divided into groups according to the concentration of bacteria (<1000 versus >1000) and the kind of growth of bacteria (positive and negative). The percentage of <1000 or >1000 bacterial numbers and positive/negative bacterial growth was compared between the four categories using the chi-square test. A result was considered statistically significant when it was P < 0.05.

## Results:

It was observed that bacterial counts regarding adhesion of bacteria at surface was 873.3±348.7, 451.1±465.3, 854.3±361.7 and 451.1±465.3 in composite resin, light cured acrylic resin, bis acryl and guttapercha respectively. The adhesion of bacteria at surface was minimum in light cured acrylic resin and guttapercha. It was maximum in composite resin and bis acryl. The bacterial adhesion in composite resin was comparable to bis acryl. Similarly, bacterial adhesion was comparable in light cured acrylic resin and guttapercha. The findings were significant statistically (Table 1).

It was also observed that bacterial microleakage was positive in 41.66% cases, 8.33 cases, 50% cases and 8.33% cases in composite resin, light cured acrylic resin, bis acryl and guttapercha respectively. Mean bacterial counts regarding bacterial microleakage were 2.6±4.8 in composite resin, 1.2±0.4 in light cured acrylic resin,2.6±4.8 in bis-acryl and 1.0±0.2 in guttapercha. These findings suggested that light cure acrylic resin and guttapercha exhibited least bacterial microleakage as compared to composite resin and bis acryl. The findings were significant statistically. There was comparable bacterial microleakage in guttapercha and light cured acrylic resin. Similarly, bacterial microleakagein composite was comparable to bis acryl (Table 2).

## Discussion:

Proper closing of the access channel is recommended in order to ensure the long-term stability and success of SRIP [12- 16]. The development of inflammation and malodour surrounding dental implants have been linked to both pathological and non-pathological bacterial microleakage, which includes the multiplication of bacteria within the implant framework [10-14]. These elements may potentially cause bone loss, which would lead to implant failure[4-8]. Studies have indicated that the use of spacer materials such as wax, cavit, vinyl polysiloxane, gutta-percha, polytetrafluoroethylene (PTFE) tape, and others was more effective than cotton pellets and endo-frost cotton pellets, which have been demonstrated to exhibit both fungal and bacterial adhesion[9-15]. According to the literature currently accessible, PTFE tape is ideal for sealing the screw channel since it carries a lower weight of microbiological volume and density, which significantly affects the long-term endurance of the implant system[16- 18]. Further investigation showed that PTFE tape was easier to handle, clean, and recover when needed [6-9].This in vitro study was carried out for evaluating and comparing bacterial adhesion and microleakage of four different materials (composite resin, light cured acrylic resin, bis acryl and Gutta-percha) utilised to seal the access passage of SRIP, while using PTFE tape as a spacer material.

There are some studies that support findings of our study showing least bacterial adhesion in Gutta-percha [24, 25, 26-27]. Our study also showed results for reduced bacterial adhesion in light cured acrylic resin. This finding is also observed in some other studies [21-26]. There are some studies; however that don't show any significant difference in bacterial adhesion among composite resin, light cured acrylic resin and bisacryl [23, 24, 25, 26-27]. This finding is not similar to findings of our study, because we found significant reduction in bacterial adhesion in light cured acrylic resin and Gutta-percha. The degree to which different materials cover the SRIP access channel has been the subject of numerous investigations [12- 18]. Most of these studies looked at the effectiveness of the inner filling materials rather than the restorative materials that can be used to cover the coronal section of the screw-access aperture [9- 16]. According to a study that measured the microbiological load that occurred when spacer materials were employed to seal the complete screw-access passage, the least number of microbial organism species were found when gutta-percha or PTFE tape was utilized in conjunction with resin composite [7-13]. The combination of cotton pellet and light-cured provisional composite, however, was associated with the highest percentage of microbial colonies [6-12].

Composite resin is currently the material most commonly used in dental restorative operations. Additionally, it has been used as the typical material to cover the SRIP access passageways' coronal section [6-9]. The superior mechanical and aesthetic properties of composite resin products are the primary drivers of their use. The primary drawback, on the other hand, is the polymerization's shrinkage and the internal stresses that follow, which could result in the formation of microcracks and bacterial microleakage [9-16]. In our study it was also observed that bacterial microleakage was positive in 41.66%cases, 8.33 cases, 50% cases and 8.33% cases in composite resin, light cured acrylic resin, bis acryl and guttapercha respectively. Mean bacterial counts were 2.6±4.8 in composite resin, 1.2±0.4 in light cured acrylic resin, 2.6±4.8 in bis-acryl and 1.0±0.2 in guttapercha. These findings suggested that light cure acrylic resin and guttapercha exhibited least bacterial microleakage as compared to composite resin and bis acryl. The findings were significant statistically. There was comparable bacterial microleakage in guttapercha and light cured acrylic resin. Similarly, bacterial microleakage in composite was comparable to bisacryl. The findings of our study similar to findings of some other studies conducted to evaluate the bacterial microleakage in different restorative materials for sealing accessory canals in SRIPs and concluded significant reduced microleakagein guttapercha [19-26].

However, some studies do not support findings of our study because they conclude no significant difference in the restorative materials regarding microleakage [20-27]. One of the materials used most frequently in prosthodontics to create a temporary fixed prosthesis is polymethyl methacrylate acrylic (PMMA) [12-19]. PMMA is a reasonably priced polymer that polishes and is marginally adaptable. The main disadvantages, however, are increased polymerization shrinkage and wear susceptibility [4-8]. Glycidyl methacrylate, sometimes known as bisphenol Bis-GMA, was later developed for similar purposes. Comparative research indicates that Bis-GMA material is superior to PMMA due to its higher marginal adaptation, less polymerization shrinkage, and decreased bacterial microleakage [18- 27]. The inorganic filler element of Bis-GMA increases its resistance to abrasion. The numerous restorative materials utilized to seal the coronal section of the SRIP access route and prevent improved bacterial penetration and proliferation are the subject of a dearth of published research [11-16].

## Conclusion:

Guttapercha and light cured acrylic resin were found to have comparatively low bacterial adhesion and bacterial microleakage in sealing accessory canals in screw retained implant supported prosthesis.

## Figures and Tables

**Table 1:** Quantitative data regarding adhesion of bacteria at surface after seven days of incubation

		Bacterial count concentration, N (%)	Bacterial counts	P value
	<1000	>1000	Mean±SD	
Composite resin	2 (16.67)	10 (83.34)	873.3±348.7	
Acrylic resin	9 (75)	3 (25)	451.1±465.3	0.001
Bis-acryl	3 (25)	9 (75)	854.3±361.7	
Guttapercha	10 (83.34)	2 (16.67)	451.1±465.3	

**Table 2:** Quantitative data of microleakage of bacteria after seven days of incubation

		Bacterial Growth, n (%)	Bacterial counts
	Negative (=0)	Positive (>0)	Mean±SD
Composite resin	7 (58.34)	5 (41.66)	2.6±4.8
Acrylic resin	11 (91.67)	1 (8.33)	1.2±0.4
Bis-acryl	6 (50)	6(50)	2.6±4.8
Guttapercha	11 (91.67)	1 (8.33)	1.0±0.2
